# Biological responses of the marine diatom *Chaetoceros socialis* to changing environmental conditions: A laboratory experiment

**DOI:** 10.1371/journal.pone.0188615

**Published:** 2017-11-30

**Authors:** Xuefeng Li, Nathalie Roevros, Frank Dehairs, Lei Chou

**Affiliations:** 1 Service de Biogéochimie et Modélisation du Système Terre - Océanographie Chimique et Géochimie des Eaux, Université Libre de Bruxelles (ULB), Brussels, Belgium; 2 Earth System Sciences & Analytical, Environmental and Geo-Chemistry, Vrije Universiteit Brussel (VUB), Brussels, Belgium; Stazione Zoologica Anton Dohrn, ITALY

## Abstract

Diatoms constitute a major group of phytoplankton, accounting for ~20% of the world’s primary production. It has been shown that iron (Fe) can be the limiting factor for phytoplankton growth, in particular, in the HNLC (High Nutrient Low Chlorophyll) regions. Iron plays thus an essential role in governing the marine primary productivity and the efficiency of biological carbon pump. Oceanic systems are undergoing continuous modifications at varying rates and magnitudes as a result of changing climate. The objective of our research is to evaluate how changing environmental conditions (dust deposition, ocean warming and acidification) can affect marine Fe biogeochemistry and diatom growth. Laboratory culture experiments using a marine diatom *Chaetoceros socialis* were conducted at two temperatures (13°C and 18°C) and under two pCO_2_ (carbon dioxide partial pressure) (400 μatm and 800 μatm) conditions. The present study clearly highlights the effect of ocean acidification on enhancing the release of Fe upon dust deposition. Our results also confirm that being a potential source of Fe, dust provides in addition a readily utilizable source of macronutrients such as dissolved phosphate (PO4) and silicate (DSi). However, elevated atmospheric CO_2_ concentrations may also have an adverse impact on diatom growth, causing a decrease in cell size and possible further changes in phytoplankton composition. Meanwhile, ocean warming may lead to the reduction of diatom production and cell size, inducing poleward shifts in the biogeographic distribution of diatoms. The changing climate has thus a significant implication for ocean phytoplankton growth, cell size and primary productivity, phytoplankton distribution and community composition, and carbon (C), nitrogen (N), phosphorus (P), silicon (Si) and Fe biogeochemical cycles in various ways.

## 1. Introduction

Diatoms are responsible for around 20% of global primary production and therefore play an important role in determining the organic C export from surface waters to the deep ocean [1]. Diatoms are dominant species in the natural phytoplankton assemblages especially in nutrient-rich ocean regions, such as continental margins and upwelling areas [2]. Marine phytoplankton growth is controlled by the availability of macro-nutrients (N, P, and Si) and is further regulated by a number of dissolved trace metals. Of these, Fe exerts an essential role in controlling the marine primary productivity and the efficiency of biological C pump, through many important biochemical processes, such as photosynthetic and respiratory electron transport, nitrogen fixation, and chlorophyll synthesis [3]. However, the concentrations of Fe in most areas of the open ocean surface are extremely low because of its limited solubility, ranging between picomolar and nanomolar levels. In oxygenated seawaters, Fe^3+^ is present as highly insoluble oxyhydroxides, while soluble Fe^2+^ is thermodynamically unstable and rapidly oxidises to Fe^3+^ [3]. This micronutrient has been shown to be the limiting factor governing phytoplankton growth and primary production over approximately 30–50% of the modern ocean, in particular, in the HNLC regions [4]. Although there remains a few controversies surrounding Martin’s “Iron Hypothesis”, Fe is considered as an important trace element which has a strong link to climate change, stimulating phytoplankton growth and increasing the drawdown of the atmospheric CO_2_ [5–9]. Therefore, Fe biogeochemistry has been one of the hot topics of oceanographic research for the past decades.

We are currently living in a world that is continuously changing. All the climate change drivers can work together and alter the ocean properties. Global warming will increase stratification of the surface waters, reducing the vertical supply of nutrients in the mixed layer. Increasing atmospheric CO_2_ will further acidify seawater and lower the calcium carbonate (CaCO_3_) saturation state which will possibly lead to a decreased calcification rate. Additionally, changes in storm activity and dust deposition will influence ocean physics and chemistry, marine organisms and ecosystems. The ocean will thus become warmer, fresher and less alkaline in the future [10]. Thus, marine biogeochemistry issues should be resolved by evaluating the impact of multiple environmental factors and in the context of global climate change, especially with modifications of seawater chemistry including dust deposition, pH and sea surface temperature.

Mineral dust is considered as one of the main source of essential nutrients, such as NO3, PO4, DSi and Fe to the open ocean, which makes the atmospheric deposition of dust-bound nutrients to the ocean and their subsequent release into seawater an important process for phytoplankton growth [7, 11–13]. Particle size of mineral dusts may vary between 0.1 to 10 μm, with a mean diameter of around 2 μm [7]. Once lifted into the atmosphere, such aerosols have long enough lifetimes, ranging from a few hours to up to weeks, allowing them to be transported from the source areas over thousands of kilometers [7, 14]. For instance, million tons of Saharan dust can be transported across the entire Atlantic Ocean to the Amazon Rainforest and the Caribbean Sea [15]. In addition, during the dust transport in the atmosphere, a number of atmospheric processes are known to strongly affect the speciation and solubility of various elements such as Fe, P and Si in dust particles [16, 17]. Atmospheric dust deposition is characterised by extreme temporal and spatial variability as well as high sensitivity to changes in climate. The robust prediction about the future shifts in global dust deposition is a major challenge, ranging from a 300% increase [18] to a 60% decrease [19] compared to current dust loading. Thus, there are still large uncertainties in the assessment of the solubility and bioavailability of nutrients upon mineral dust deposition and its impact on phytoplankton growth especially under changing climate.

Global warming gave rise to an increase by 0.74±1.18°C in global average surface ocean temperature from 1906 to 2005 [20]. Under the Representative Concentration Pathway (RCP) 8.5 scenario, the surface seawater in 2100 will be warmer by more than 4°C compared to that in 1950 [9]. All marine organisms have limiting or optimal temperature ranges [21]. As a key determinant interacting with numerous other abiotic and biotic factors, temperature defines the geographic range of many species. Therefore, global warming could lead to shifts in the geographical distribution of marine species and cause further changes in community composition and oceanic biodiversity [9, 22]. This has a profound impact not only on the vertical stratification of the water column which will increase, but also on the supply of dissolved Fe and other nutrients to the surface waters from deep ocean which will decrease [23].

It is estimated that seawater pH will decrease from an original value of 8.25 since the onset of the industrial revolution to an estimated value of 7.85 by the end of this century and this value will go down further by up to 0.7 units by the year 2300 [24]. Ocean acidification is the term given to the ongoing phenomenon that shows a decrease in seawater pH, caused by the uptake of anthropogenic CO_2_ from the atmosphere [24, 25]. A number of long-term observations, direct measurements and future model scenarios have provided evidence that rising atmospheric CO_2_ level will continue to contribute to ocean acidification, rendering the ocean even less alkaline [26–28]. Ocean acidification is likely to affect marine ecosystems, but recent studies on a broad range of marine organisms show a considerable variation in their vulnerability and tolerance [29–31].

Oceanic systems have been affected and will continue to be influenced by changing environmental conditions under various rates, magnitudes and durations [9]. In the present study, the main objective is to evaluate how global change processes, in particular, dust deposition, sea-surface warming and ocean acidification, could affect diatom growth and nutrient biogeochemical cycles. To achieve these aims, laboratory culture experiments were conducted on a major oceanic phytoplankton group (diatoms) *Chaetoceros socialis*, under controlled conditions (temperature, light intensity, pCO_2_, and dust input).

## 2. Materials and methods

### 2.1 Cultures and media preparation

The cosmopolitan, centric and chain-forming diatom *Chaetoceros socialis* was originally described by Lauder [32] from waters adjacent to Hong Kong (South China Sea). Monospecific *C*. *socialis* (isolated from the Belgian Coastal Zone, 2012) was cultivated in 0.2 μm filtered and sterilised North Atlantic seawater (48° 48.9 ‘N and 10° 24.7’ W) which had a salinity of 35.59. This seawater was enriched with dissolved nitrates (NO3), phosphates (PO4) and silicate (DSi) to achieve final concentrations of 88, 4 and 11 μmol L^−1^, respectively. This nutrient mix corresponds to the f/20 medium [33]. For trace metals and vitamins, the f/2 standard recipe was followed. The dissolved Fe concentration in the medium was 0.3 μmol L^−1^. All labware of the set-up for culturing (incubation bottles, caps, tubes and sampling syringes *etc*.) were made of Teflon or polycarbonate, and thoroughly acid cleaned and sterilized prior to the experiments to avoid any potential trace-metal contamination.

### 2.2 Experimental design and sampling protocol

Laboratory batch culture experiments were performed at two temperatures (13°C and 18°C) and two pCO_2_ corresponding to present-day (400 μatm) and near-future (800 μatm) conditions. In order to test all possible combinations of these variables, four pCO_2_/temperature treatments were examined in duplicate: (i) Present-day treatment: 13°C and 400 μatm pCO_2_; (ii) High temperature (ocean warming) treatment: 18°C and 400 μatm pCO_2_, with only the increase of temperature; (iii) High pCO_2_ (ocean acidification) treatment: 13°C and 800 μatm pCO_2_, with only the increase of pCO_2_; and (iv) Greenhouse treatment: 18°C and 800 μatm pCO_2_, with both the increase of temperature and pCO_2_, simultaneously.

The 10L Nalgene polycarbonate bottles containing the strain and medium were placed in a temperature-controlled incubator at constant incident photon flux density (150 μmol m^−2^ s^−1^) with a 14h/10h light/dark cycle. Initial cell density in the culture experiment was controlled at 500 cell mL^-1^. Specific growth rates (μ) were calculated as the slope of a linear regression of the natural logarithm of cell concentration against time over the exponential growth phase for each treatment. For each treatment, there was a treatment without the addition of dust and duplicates with addition of 100 mg L^-1^ desert dust (<63 μm) after the maximum growth was reached. Based on the frequent measurements during the exponential phase period, dust particles were added once the florescence value had reached its maximum. During the growth stage of the experiments, various parameters were monitored: pH, alkalinity, pCO_2_, fluorescence, turbidity, cell abundance, chlorophyll-a (Chl-a), nutrients (NO3, PO4 and DSi), dissolved Fe, biogenic silica (BSi), particulate organic carbon and nitrogen (POC/PN). BSi was not measured after the dust addition because of the presence of important lithogenic silica in mineral dust particles added. In general, sampling frequency was depending on the growth phase of the culture. Daily sampling was done during the exponential phase, and also every two or three days during the lag and decline phase. Samples were directly taken from the incubation bottles with a sterile syringe, always at the same time of the day in the light cycle, after gentle shaking of the incubation bottles.

Samples for the analyses of dissolved nutrients and Fe speciation were filtered through 0.4 μm pore size Nuclepore filters and stored in 15-mL centrifuge tubes. For NO3 and PO4, the filtrates were kept frozen until analysis. For DSi, the filtrates were acidified to a pH value close to 3 and stored at 4°C. For dissolved Fe analyses, the filtrates were acidified to a pH value close to 1 and kept at 4°C until analysis. Samples of 40 mL for chl-a measurement were filtered through 25 mm Whatman GF/F glass fibre filters, and the filters were stored frozen in centrifuge tubes wrapped with aluminium foil prior to analysis. Samples for BSi analysis were filtered onto 0.4 μM 25 mm polycarbonate filters and the filters were kept in a petri dish in a freezer until analysis. Samples for POC/PN analyses were collected by filtration onto pre-combusted (500°C, 4h) Whatman GF/F glass fibre filters and kept frozen in a petri dish until analysis. Before the analysis, the filters were exposed to strong HCl fumes overnight to remove the carbonates on the filters.

### 2.3 Desert dust

Desert dust particles (<63 μm) used in our experiments were sampled from the Kubuqi Desert (40°27’ N, 108°38’ E), part of the Gobi desert, north of the Erdos Plateau in Inner Mongolia Autonomous Region, China in November 2012. The Gobi desert is one of the major source regions of East Asian dust [34]. Particularly in each spring and early summer, massive surface dusts from the Gobi desert region travel over China, the Korean Peninsula, and Japan into the downwind seas, including the China seas, the Sea of Japan, the subarctic North Pacific, the North Pacific subtropical gyre, and the western and eastern Equatorial Pacific [35, 36].

The desert surface is covered by fine sand, with more than 81% of the particles ranging between 100 μm and 250 μm in size, and with only 2% smaller than 63 μm [37]. Dust particles were collected through 2 sieves (acid clean), first with the size fraction less than 150 μm then less than 63 μm. Further sieving became extremely difficult and time-consuming. Consequently, in our study, the dust particle size of <63 μm was used in the culture experiments. It can be expected that larger particles would fall out of the atmosphere too rapidly to be transported over long distances. However, atmospheric transport of mineral dusts of >63 μm has been proposed to occur more often than previously thought and these particles can be carried over longer distances [38].

The amount of dust that we added in our experimental represented a high dust loading, corresponding to 50 times higher than dry dust deposition [39] and 10 times higher than the desert rain event [40]. But the addition was still acceptable [41], since Saharan dust deposition events could carry between 5 and 8000 mg L^-1^ dust in the rain [40].

X-ray diffraction analysis shows that the dust consists mainly of quartz, calcite and plagioclase (disordered albite). Determination of the concentrations of particulate major, minor and trace elements indicates that the dust particle contains about 30.8% Si, 3.57% Al, 2.58% Fe, 0.19% P and 0.14% organic C. For each treatment, a dust dissolution control test without diatoms was conducted in culture medium under the same conditions in order to evaluate the amount of nutrients released from the dust.

### 2.4 Analytical methods

**Fig 1:**
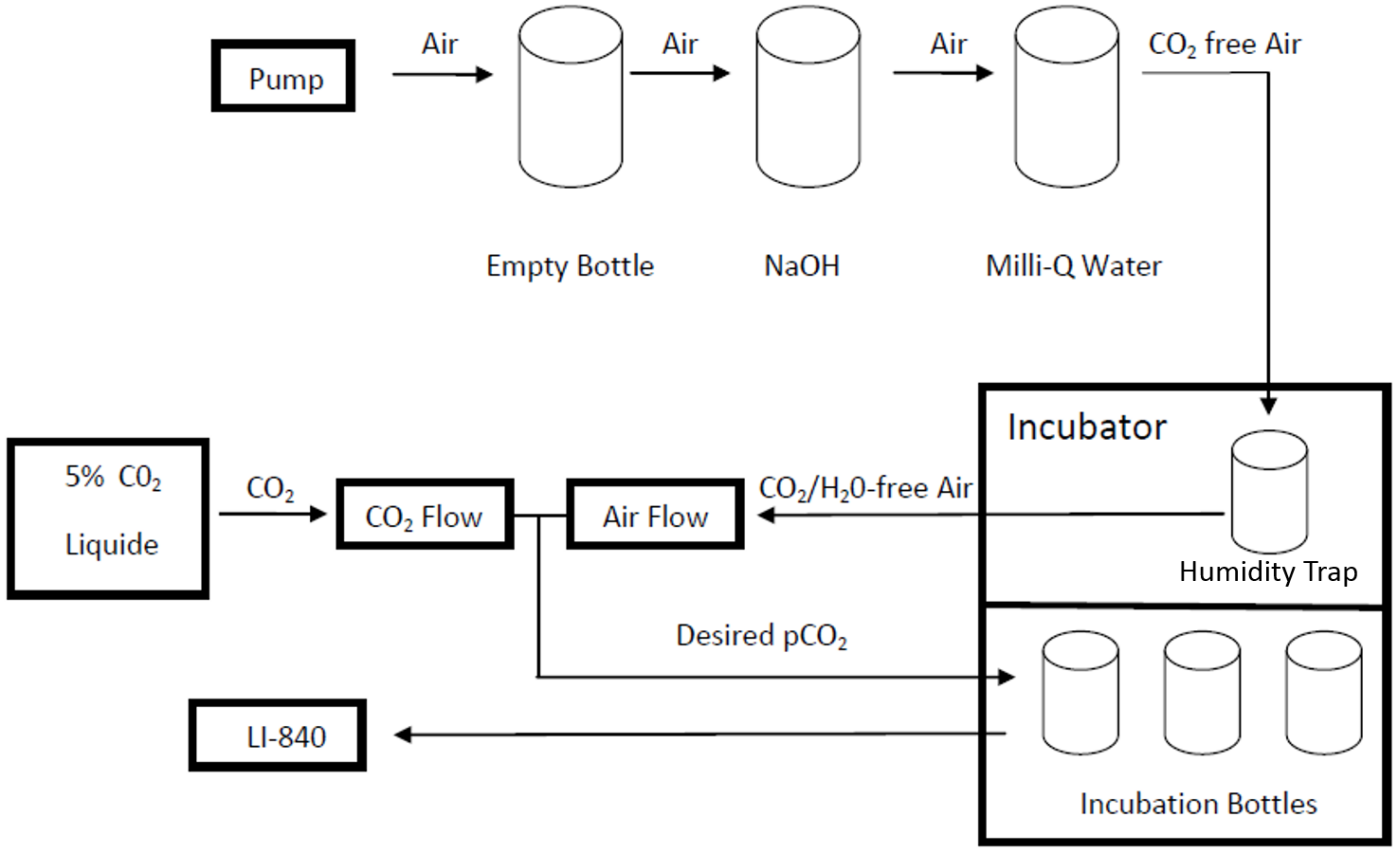
Schematic representation of the CO_2_ controlling system in the culture experiments. The pumped ambient air went through an empty safe bottle (preventing the reversal flow of the NaOH solution from entering the pump system), a bottle containing 1M sodium hydroxide (NaOH) solution (absorbing CO_2_ in the air flow), a bottle with Milli-Q water (removing any residual NaOH solution in the moisture), and a humidity trap bottle (condensing and dehumidifying the air), successively. Then CO_2_/H_2_O-free air was mixed with a gas mixture of CO_2_/O_2_ containing 5% CO_2_. A CO_2_/H_2_O gas analyzer (LI-840) was connected to the bubbling system and used to verify the desired pCO_2_ concentration into the culture medium. The flow rate of the 5% CO_2_ was re-adjusted whenever the pCO_2_ deviated from the value initially set for each treatment.

A desired pCO_2_ was obtained by continuous gentle bubbling of CO_2_-free ambient air flow mixed with 5% pure CO_2_ (Air Liquide, Belgium) during the entire experiment. A CO_2_/H_2_O gas analyzer (LICOR 840) was connected to the bubbling system and used to check pCO_2_ concentration in the incubation bottles throughout the experiments (Fig 1). Total Alkalinity (TA) was measured by potentiometric titration with HCl (0.1 N, Merck) using the Gran procedure [42]. The measurement of pH was made with a combined pH electrode (Metrohm) using TRIS and AMP buffers prepared according to Dickson *et al*. [43]. The parameters of the carbonate system were calculated based on the measurement of TA, pCO_2_, temperature, salinity and nutrients concentrations using the CO2SYS programme [44].

Fluorescence and turbidity were determined with a Turner fluorometer-turbidimeter. And fluorescence values were used as an indicator for diatom growth. Cell abundance (number of cells per mL) was examined by haemocytometer counting plates using a light microscope. Chl-a concentrations were measured following the fluorimetric method of Yentsch and Menzel [45] using a Shimadzu RF-150 fluorometer with an excitation wavelength of 430 nm and an emission wavelength of 663 nm.

Dissolved NO3 was measured colorimetrically using a Skalar Autoanalyzer system [46]. Concentrations of PO4 and DSi were determined manually with a spectrophotometer at a wavelength of 880 nm and 810nm, respectively [46]. The drawdown ratios of ΔN/ΔP and ΔN/ΔSi were calculated from the changes in NO3, PO4 and DSi between two successive sampling days during the exponential growth phase. Dissolved Fe concentrations were spectrophotometrically determined at 562 nm, following the ferrozine methods of Viollier *et al*. [47].

Elemental Analyzer—Isotope Ratio Mass Spectrometry (EA-IRMS) was used to measure the POC/PN concentrations. The concentration of BSi produced was determined during the exponential growth phase, following the method described by Carbonnel *et al*. [48].

### 2.5 Statistical treatment of data

The influence of dust addition on biological data (Chl-a concentration and cell number) and on other parameters (NO3, PO4, DSi, dissolved Fe and POC/PN) was evaluated with a t-test. A one way or two-way analysis of variance (ANOVA) was used to identify the statistically significant impact of the different CO_2_ pressures (400 μatm and 800 μatm) and temperatures (13°C and 18°C). All statistical treatments of data were performed using the SPSS 19.0 software. The confidence level for all analyses was set at 95%.

## 3. Results

### 3.1 Carbonate chemistry system

The controlled pCO_2_ and temperature set for each treatment remained stable during the entire incubation experiments, and approached closely to the target values (Table 1). There were however some fluctuations in pCO_2_ values for the High pCO_2_ and Greenhouse conditions, which could be largely ascribed to biological activities and variable ambient air flow. Measured and calculated parameters of carbonate chemistry in the culture media are summarized in Table 1. With TA kept relatively constant, a higher pCO_2_ would lead to increased dissolved inorganic carbon (DIC) and bicarbonate ion (HCO_3_^-^) concentrations whereas it would decrease the pH and carbonate ion (CO_3_^2-^) concentration, conditions which were well representative of ocean acidification toward the end of this century.

**Table 1:** Carbonate system parameters in the four pCO_2_/temperature treatments.

Treatments	T	CO_2_	pH	TA	DIC	HCO_3_^-^	CO_3_^2-^
	°C	μatm		μmol kg^-1^	μmol kg^-1^	μmol kg^-1^	μmol kg^-1^
**Present-day**	13	401±15	8.03±0.01	2288±20	2082±22	1918±23	148±3
**High temperature**	18	395±12	8.04±0.02	2319±71	2067±61	1874±51	179±11
**High pCO**_**2**_	13	758±28	7.78±0.02	2269±27	2161±21	2043±19	87±4
**Greenhouse**	18	836±16	7.75±0.01	2296±37	2173±32	2044±17	101±7

The measured values parameters include temperature, pCO_2_ and TA and pH. DIC, HCO_3_^-^ and CO_3_^2-^ concentrations were calculated using the CO2SYS programme, based on the pCO_2_ and TA measurements. The values were means ± standard deviation, averaged from several measurements throughout the experiment. The number of measurements differ between the different treatments, but basically, they exceed 10.

### 3.2 Effects of dust addition

**Fig 2:**
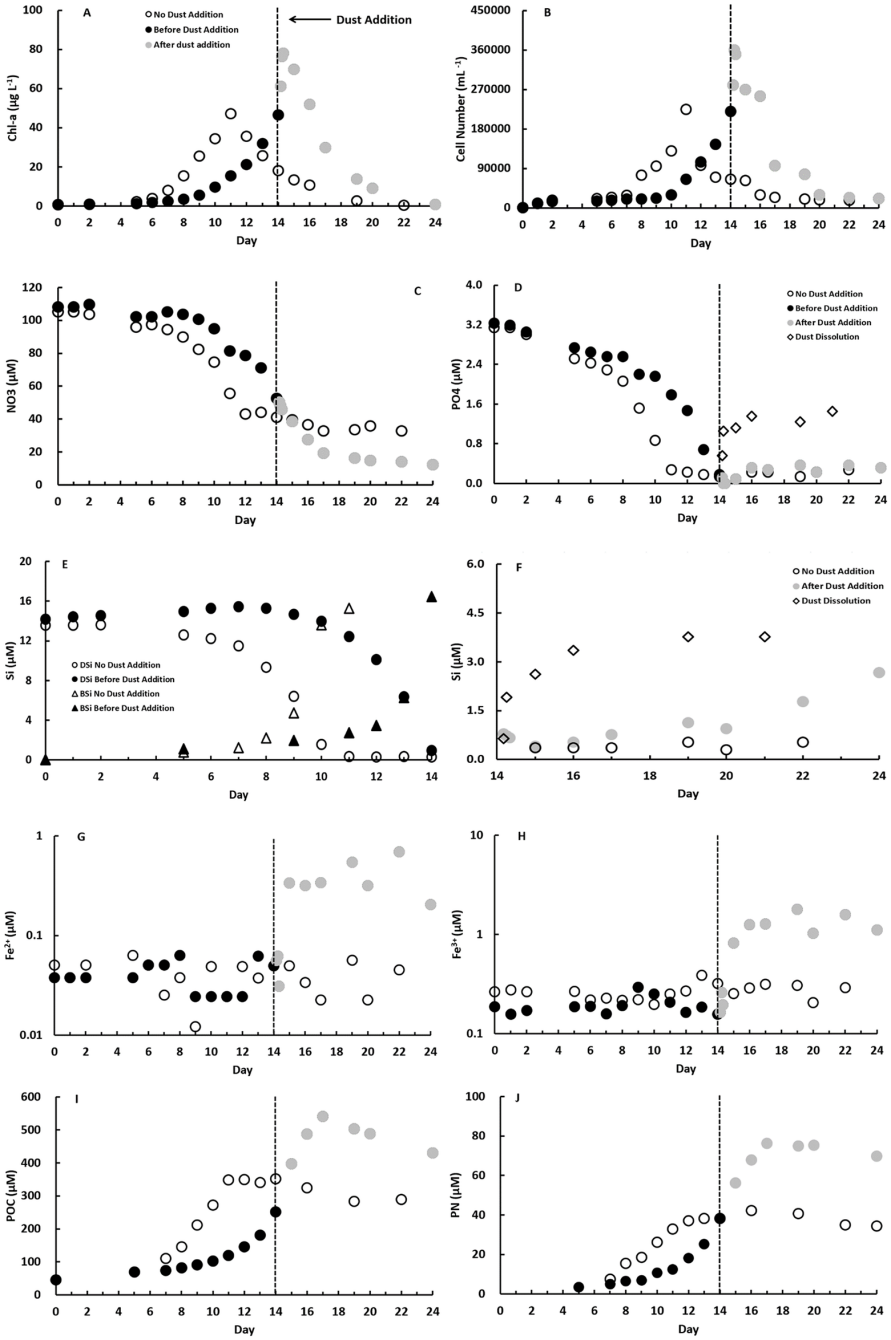
Evolution of diatom growth and nutrient concentrations during the culture experiments at 400 μatm pCO_2_ and 18°C. (A) chlorophyll-a, (B) cell number, (C) NO3, (D) PO4, (E) DSi and BSi before dust addition on day 14 (reaching maximum growth), (F) DSi after dust addition on day 14, (G) dissolved Fe^+2^, (H) dissolved Fe^+3^, (I) POC and (J) PN for both treatments, without and with dust addition. No dust addition treatment, before and after dust addition treatments and dust dissolution test are shown respectively as open circles, closed black and grey circles, and open diamonds. Vertical dashed lines indicate the day on which the dust (100 mg/L) was added to the dust treatment bottle. For (E), open and close triangles denote BSi concentrations in no dust treatment and dust treatment, respectively.

For the sake of clarity, only the results of one of the duplicates from High Temperature treatment (400 μatm pCO_2_ + 18°C) are presented here as an example to compare the impacts of dust particle addition on diatom growth (Fig 2). Because the duration of the lag phase for diatom growth between treatments was different even in duplicates, it was impractical to average the results of the duplicates. But the duplicates well presented the same growth patterns and biological responses to dust addition. Similar trends were also obtained for all the other pCO_2_ and temperature treatments (S1–S3 Figs).

During the initial diatom growth, Chl-a concentration and cell number increased for both treatments, without and with dust addition, under high temperature condition (18°C), which was accompanied by a decrease in macronutrients concentrations (NO3, PO4 and DSi) and an increase in BSi, POC and PN concentrations (Fig 2A–2J). For the treatment without dust addition, Chl-a and cell abundance increased till a maximum value was reached on Day 11 (47.1 μg L^-1^, and 22500 cells mL^-1^, respectively) (Fig 2A and 2B). Soon afterwards, all growth parameters (Fig 2A and 2B) began to decrease corresponding to PO4 and DSi depletion (Fig 2D and 2E). For the dust addition treatment, once Chl-a and cell number reached its maximum value (46.5 μg L^-1^, and 22000 cells mL^-1^, respectively) on Day 14 (3-day lag compared to no dust addition treatment), dust particles were added and significant increases were immediately observed for Chl-a (up to 78.0 μg L^-1^) and cell abundance (up to 36000 cells mL^-1^) representing increases of 68% and 64%, respectively (Fig 2A and 2B). The results clearly demonstrate that dust addition stimulated diatoms C. *socialis* growth (even though inducing a lower light intensity). Higher biological POC and PN concentrations after dust addition also confirmed this observation (Fig 2I and 2J), as these increased respectively by 54% and 80% relative to the treatment without dust addition. It should be noted that POC and PN concentrations measured in our treatment may include also detrital organic matter originated from the dust added. However, the detrital organic carbon added was not significant because the dust contained only 0.14% POC. The different changes reflecting enhanced biological activity were statistically significant as well. After the addition of dust particles, all the biological variables (Chl-a, cell abundance, POC and PN) show a significantly increase (t-test, all p<0.05).

From the beginning of the exponential growth phase, NO3, PO4 and DSi were consumed to support diatom growth (Fig 2C–2E). At the same time, the results show that DSi was roughly quantitatively transformed into BSi (Fig 2E). Because the lithogenic source from dust particles would contribute the pool of particulate Si after dust addition, BSi was determined only for the exponential growth phase before dust addition. Soon after the dust particles were added on Day 14, we observed a slight decrease in NO3, which was not statistically significant (Fig 2C, t-test, p>0.05), no significant difference in PO4 concentrations (Fig 2D, t-test, p>0.05), but a significant increase in DSi concentrations (Fig 2F, t-test, p<0.05), compared to the treatment without dust addition. In order to evaluate the amount of nutrients released from the dust, a dust dissolution control test was also conducted under the same conditions of pCO_2_ and temperature. The results show that the amount of PO4 and DSi dissolved from the dust (up to 1.5 μM and 3.8 μM respectively) were immediately consumed by the diatoms, since the nutrient concentrations (PO4 and DSi) in the growth medium remained as low as those for the treatment without dust addition (Fig 2D and 2F). The difference between nutrient concentration in the dust dissolution experiment and the nutrient level in the growth media thus corresponded to the amount of extra nutrients consumed by diatoms, which were of dust origin. Contrary to PO4 and DSi, we did not detect a release of NO3 from the dust; the NO3 concentration continued to decrease and thus was consumed. During the diatom growth, dissolved Fe concentrations for both Fe^2+^ and Fe^3+^ remained stable and no significant consumption was detected. After dust addition, dissolved Fe concentrations increased significantly, for both Fe^2+^ and Fe^3+^, from 0.05 μM to 0.69 μM and from 0.16 μM to 1.78 μM for Fe^2+^ and Fe^3+^, respectively (Fig 2G and 2H).

### 3.3 Effects of ocean warming and acidification

As a general feature, fluorescence, Chl-a and cell number increased and BSi, POC and PN accumulated, while nutrients (NO3, PO4 and DSi) were consumed during the course of the growth phase for each treatment. After addition of dust material, fluorescence, Chl-a, cell number, POC and PN all significantly increased, compared to the treatment without dust addition in which the bloom collapsed once the nutrients became exhausted. Similar to the results shown in the previous section, PO4 and DSi released from dust dissolution were then consumed immediately. There was no NO3 released, while much higher dissolved Fe concentrations, for both Fe^2+^ and Fe^3+^, were found after dust addition (Fig 2 and S1–S3 Figs).

**Fig 3:**
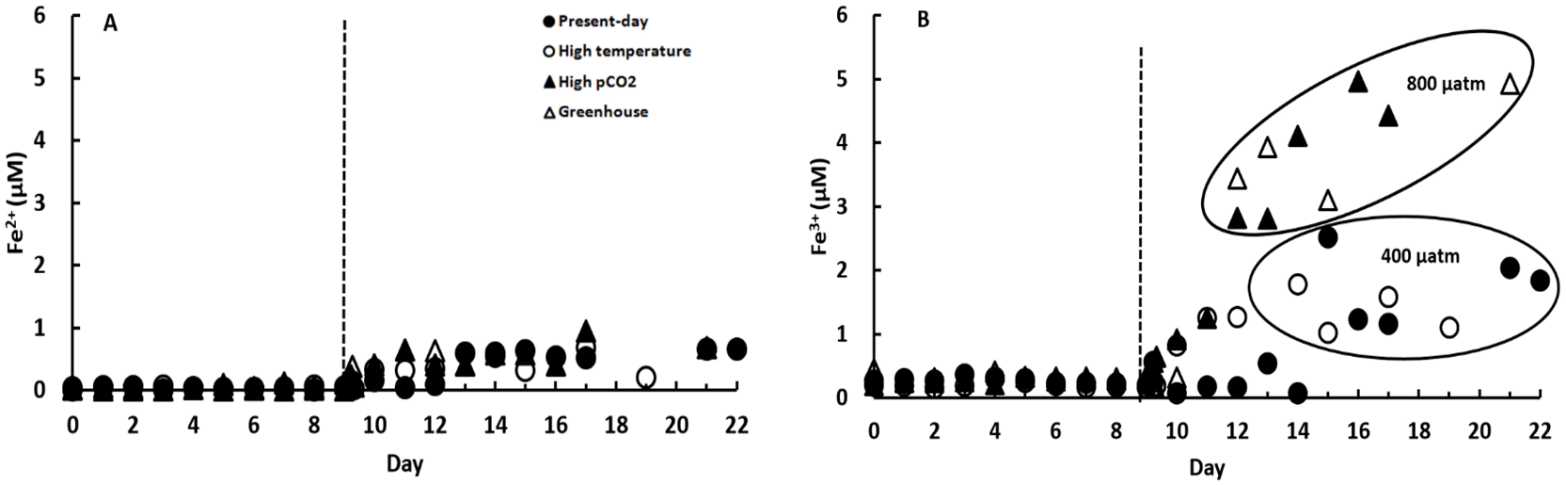
The evolution of (A) dissolved Fe^2+^ and (B) dissolved Fe^3+^ for the four treatments. Four treatments correspond to Present-day (400 μatm pCO_2_ and 13°C), High Temperature (400 μatm pCO_2_ and 18°C), High pCO_2_ (800 μatm pCO_2_ and 13°C), and Greenhouse (800 μatm pCO_2_ and 18°C), respectively. Vertical dashed lines indicate the day on which the dust (100 mg/L) was added.

There was no significant variation in the NO3, PO4 and DSi consumption during the growth stage among the 4 treatments (Fig 2C–2E and S1C–S1E, S2C–S2E and S3C–S3E Figs, one-way ANOVA, p>0.05). However, for dissolved Fe, markedly higher amounts, in particular Fe^3+^, were released for High pCO_2_ and Greenhouse treatments as a result of the higher pCO_2_ and therefore lower pH of the culture media (Fig 3A and 3B, a one-way ANOVA, p<0.05). The temperature did not play a very important role in dissolved Fe release from the dust (a one-way ANOVA, p>0.05). The maximum Fe^3+^ concentration measured after dust addition was 4.93 μM for Greenhouse treatment and 4.97 μM for High pCO_2_ treatment, corresponding to pH close to 7.75. They were more than two-fold higher than those for the Present-day (2.04 μM) and High temperature treatments (1.78 μM), corresponding to pH close to 8.04. These findings indicate that ocean acidification can enhance significantly the availability of Fe upon dust deposition, which is not surprising.

**Fig 4:**
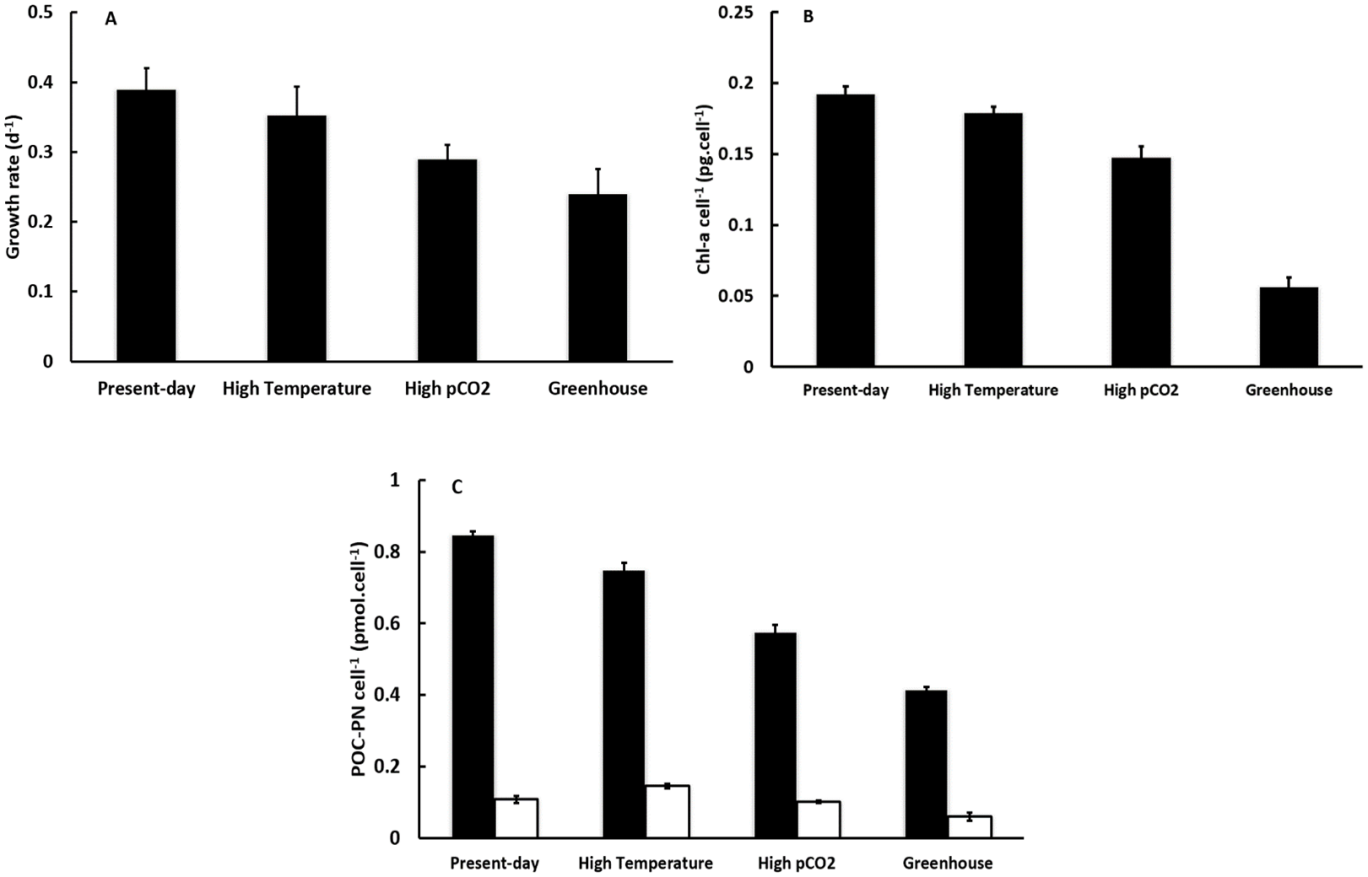
Results of (A) growth rate, (B) cellular chl-a content, (C) cellular POC and PN contents for the exponential growth phase of the four treatments before dust addition. In (C), filled bars indicate the cellular contents of POC and open bars those of PN. Four treatments represent Present-day (400 μatm pCO_2_ and 13°C), High Temperature (400 μatm pCO_2_ and 18°C), High pCO_2_ (800 μatm pCO_2_ and 13°C), and Greenhouse (800 μatm pCO_2_ and 18°C), respectively.

The averaged values of growth rate, cell abundance normalised Chl-a content, and POC/PN contents were calculated from data obtained during the exponential growth phases preceding dust addition from duplicate cultures. The highest growth rate, cellular Chl-a and POC content were found for the Present-day treatment (400 μatm pCO_2_ and 13°C), while the lowest growth rate, cellular Chl-a, cellular POC and PN contents were observed for the Greenhouse treatment (800 μatm pCO_2_ and 18°C) (Fig 4A–4C). The growth rate under the Present-day condition was 0.405 d^-1^, which was 7%, 28%, 40% higher than that determined for the High temperature, High pCO_2_ and Greenhouse treatments, respectively (Fig 4A). The cellular Chl-a content was 0.189 pg cell^-1^, which was 7%, 23%, 71% higher than that for Higher Temperature, Higher pCO_2_ and Greenhouse treatments (Fig 4B), respectively. The cellular POC content was 0.848 pmol cell^-1^, which was 12%, 32%, 52% higher than that for the High temperature, High pCO_2_ and Greenhouse conditions, respectively (Fig 4C). Statistically, pCO_2_ had a significant negative effect on all growth parameters (growth rate, cellular Chl-a, cellular POC and PN contents, a two-way ANOVA, p<0.05). Temperature played an important role in setting the cellular Chl-a and cellular POC contents (a two-way ANOVA, p<0.05), but this was not the case for the growth rate nor the cellular PN content (a two-way ANOVA, p>0.05). A two-way ANOVA indicates a significant synergistic influence of pCO_2_ and temperature on cellular Chl-a, POC and PN contents (p<0.05), but not on growth rate (p>0.05). Thus, both high temperature and high pCO_2_ did not appear to favor the growth of diatom C. *socialis*.

**Fig 5:**
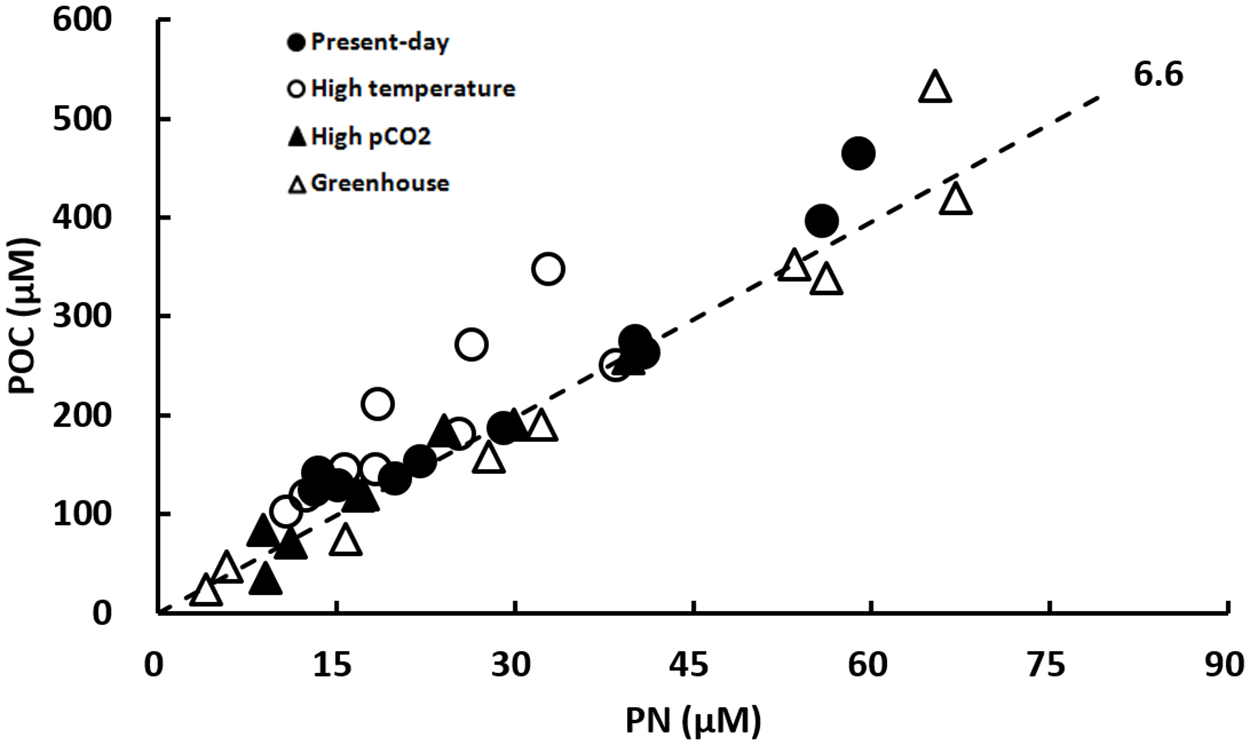
POC/PN ratios of the organic matter produced during the exponential growth phase for the four treatments before the dust addition. Four treatments are Present-day (400 μatm pCO_2_ and 13°C), High Temperature (400 μatm pCO_2_ and 18°C), High pCO_2_ (800 μatm pCO_2_ and 13°C), and Greenhouse (800 μatm pCO_2_ and 18°C), respectively. The dashed line corresponds to a C:N molar ratio of 6.6.

POC/PN ratios of the organic matter produced during the exponential growth phase are shown for the four treatments in Fig 5. They varied from 4.8 to 11.4 with an averaged value of 7.6 ± 1.7, which was fairly close to the empirical value of Redfield C:N ratio of 6.6 for phytoplankton and there was no significant difference in C/N ratio between treatments (a one-way ANOVA, p>0.05).

**Fig 6:**
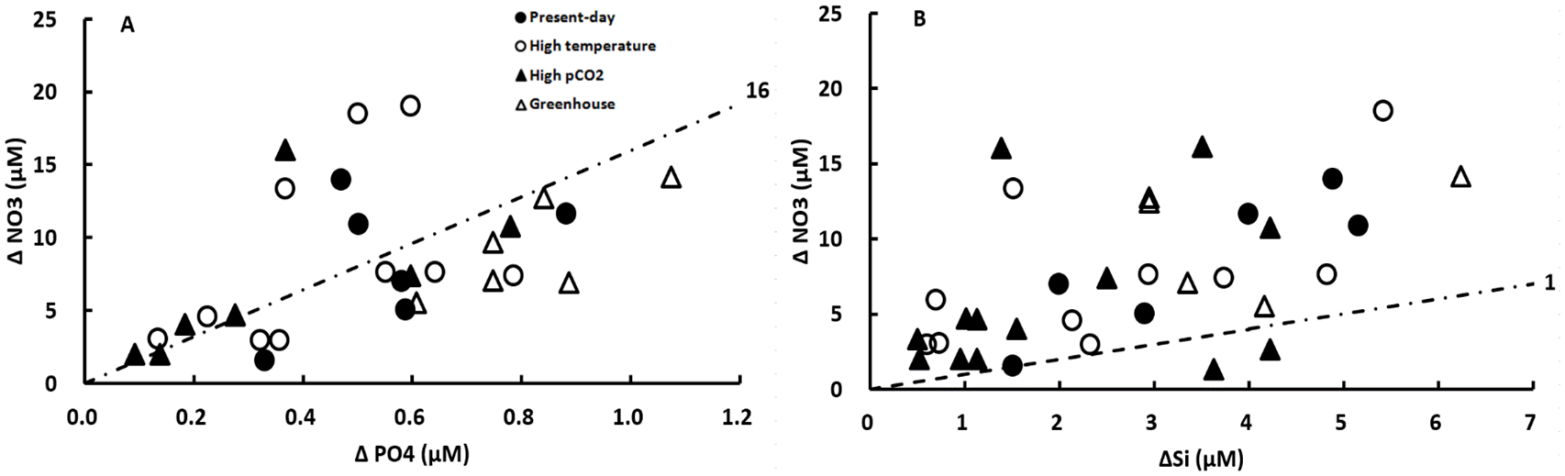
Nutrient drawdown ratios of (A) ΔN/ΔP and (B) ΔN/ΔSi during the exponential growth phase for all four treatments before the dust addition. Four treatments correspond to Present-day (400 μatm pCO_2_ and 13°C), High Temperature (400 μatm pCO_2_ and 18°C), High pCO_2_ (800 μatm pCO_2_ and 13°C), and Greenhouse (800 μatm pCO_2_ and 18°C), respectively. The dashed line indicates (A) a N:P molar ratio of 16 and (B) a N:Si molar ratio of 1.

The results for the nutrient drawdown ratios of ΔN/ΔP and ΔN/ΔSi during the exponential growth phase for the four treatments are shown in Fig 6. No significant difference was observed among treatments for both ΔN/ΔP and ΔN/ΔSi ratios (a one-way ANOVA, p>0.05). The mean ΔN/ΔP drawdown ratios were 17.4 ± 8.9 close to Redfield N:P ratio (16) but with a large variation. The mean ΔN/ΔSi drawdown ratios were 3.4 ± 2.4.

## 4. Discussion

### 4.1 Dust deposition

As an example of dust addition experiment we consider the one with 400 μatm pCO_2_ + 18°C conditions which clearly reveals that desert dust addition can significantly promote diatom *C*.*socialis* growth (Fig 2A and 2B) and biomass (Fig 2I and 2J), compared to the treatment without dust addition. Extensive laboratory experiments, on-site observations, and numerical simulations have already associated atmospheric dust deposition with increasing chlorophyll concentrations and net community production in the world oceans, indicating that atmospheric deposition can stimulate phytoplankton growth [49–51]. In the North Pacific, the passage of an Asian Gobi desert dust storm could double the phytoplankton biomass and enhance POC production [49]. In the Northeast Atlantic, Duarte *et al*. [50] observed a sharp increase in phytoplankton biomass and primary production as well as a potential impact on phytoplankton community composition with high rates of atmospheric nutrient inputs. Cassar *et al*. [51] reported that dust deposition resulted in an enhanced export production and an increased gross primary production over a large area of the Southern Ocean downwind of substantial dust source regions.

The reason why dust deposition can enhance phytoplankton activities lies in the release of biologically important trace metals (Fe) and macronutrients (NO3, PO4 and DSi) from dust particles [7, 11–13]. Iron has long been identified as a limiting nutrient in HNLC regions (equatorial Pacific Ocean, subarctic Pacific Ocean and the Southern Ocean) [3, 7]. Nitrogen often limits productivity in much of the surface oceans at low latitudes, where subsurface nutrients supply is relatively low [52]. Phosphorus availability tends to limit production in subtropical waters of the North Pacific and North Atlantic [53]. Silicon may limit diatoms growth in northern Subantarctic and northwest Atlantic waters [4]. However, two or more nutrients may often co-limit marine phytoplankton in oceanic systems. For instance, phytoplankton productivity appears to be co-limited by N and P in the oligotrophic subtropical North Atlantic Ocean [54]. Mills *et al*. [55] found Fe and P co-limitation of N_2_ fixation in the eastern tropical North Atlantic. Hoffmann et al. [56] observed that Fe and Si availability co-limited the growth of three Southern Ocean diatom species. Consequently, spatial and temporal changes in dust deposition of macro- and micro-nutrients could shift patterns of nutrient limitation and set important controls on nutrient biogeochemistry and marine microbial ecology.

The average Fe content of the Earth’s crust is 3.5% which is widely used in assessing global-scale Fe inputs to the ocean. The global average Fe solubility in aerosols is around 1–2%, and the range reported in the literature varies from 0.01 to 80% [57]. The wide difference demonstrates their high heterogeneity in size or type and large spatial and temporal variation in oceanic and atmospheric systems. In global ocean biogeochemical models, the mineral Fe solubility is set constant with values varying between 1% and 10% for the present era [58]. Our Kubuqi desert dusts contain 2.58% of Fe and the dust dissolution experiments reveal that Fe solubility is around 4% with a dust load of 100 mg/L at 400 μatm pCO_2_. After dust particles were added, the release of both Fe^2+^ and Fe^3+^ was observed, especially for Fe^3+^ (Fig 3A and 3B). The solubility of Fe in our desert dust samples falls in the range of previous measurements and model estimations. However, soil or desert sources of dust generally have a lower solubility of Fe (< 0.5%) compared to remote marine aerosols [59]. It is possible that our dust samples contain other source materials. The potential sources of soluble aerosol Fe are anthropogenic emissions, including biomass burning and fossil fuel combustion from surrounding heavily industrialized areas. Such aerosols have a higher solubility (between 2% and 19%) compared to the aluminosilicate component of mineral dust (only approximately 0.4%) [60, 61].

The fraction of P in crustal materials is very low, ranging between 0.1% and 0.2% by weight [62]. Similarly, P solubility reported in the literature is also highly variable in space and time and ranges between 2.3% and 80% [40, 63, 64]. In global deposition simulations, a P abundance of 0.105% and a solubility of 15% have been considered for dust particles [13, 65]. The desert dust used in our study comprises 0.19% of total P. After dust addition, a rapid dissolution of PO4 was observed in the first 6 hours (Fig 2D). This release continues during the following 2 days. We calculate that the P solubility in our study is around 25% with a dust concentration of 100 mg/L. This value agrees well and is consistent with the solubility reported for the desert dust in the literature [63, 66].

Silicon is one of the most abundant constituents of soil-derived mineral aerosols [67]. Crustal Si content ranges between 19% and 44% by weight depending on mineral composition. Only a small fraction of the deposited Si dissolves in seawater. Recent studies on the atmospheric input of Si to the oceans have assumed a solubility in the range 5–10% [68]. In their modelling studies, Duce *et al*. [62] and Krishnamurthy *et al*. [13] considered the dust containing a constant 30.8% of Si by weight, with a solubility of 7.5%. However, the presumed aerosol Si solubility of 7.5% may be too high. Baker *et al*. [64] measured a much lower Si solubility of the Saharan dust (0.02–1.1%). Our Gobi desert dust contains 30.8% of Si, which is present mainly in the form of quartz and albite. With the addition of 100 mg/L dust particles, a significant increase of DSi concentration up to 3.8 μM was found (Fig 2F). In our dissolution test, Si solubility is very low 0.5% as quartz is one of the major minerals detected in our dust, but this value is in agreement with those obtained for Saharan dust [64]. Other dust materials with high Si solubilities are likely to have experienced the removal of insoluble quartz particles and possible chemical transformation during atmospheric transport [13].

Nitrogen inputs to surface oceans through dust deposition also have important impacts on ocean biogeochemistry especially for the N-limited regions and non-diazotrophic organisms [69]. Contrary to the supply of Fe, P and Si, which are fundamental constituents of dust particles, N may or may not be directly bound to dust [70]. Meanwhile, the dust particles rich in carbonates can interact with atmospheric N compounds (nitric acid, nitrate, ammonia and ammonium), resulting in coating and enrichment of dust by N [71]. The interaction of airborne mineral dust with N compounds and the formation of secondary aerosol particles make atmospheric dry and wet deposition a significant source of N to the ocean [72]. Our desert dust samples were collected directly from the desert surface, which had probably gone through little atmospheric processing since there was no apparent soluble NO3 released from the dust particles (Fig 2C).

Desert dust deposition has the potential to modify oceanic productivity predominantly through the supply of various nutrients to stimulate phytoplankton growth, although the key nutrients in each case may be different. The type and solubility of supplied nutrients depend on the physicochemical properties of a dust sample, for instance, the chemical composition, minerology and sources *etc*., as well as the atmospheric chemical and photochemical processing function during the transport [7, 11, 73]. It should be noted that in our experiments the addition of Gobi desert dust could have enhanced diatom growth, mainly through the release of dissolved PO4 and Si rather than Fe (Figs 2D–2H, 3A and 3B). Just before the dust particles were added, both dissolved Si and PO4 were depleted in our culture medium, but this was not the case for Fe nor for NO3. Dust addition could indeed provide more dissolved Fe to the medium, but that was not the main reason why the growth was stimulated in our case. Since the starting concentrations of dissolved Fe were relatively high, Fe was not the limiting factor throughout culture experiments.

### 4.2 Ocean warming and acidification

In our culture experiments on diatom *C*. *socialis*, the results indicated negative biological responses to increased temperature. At 18°C, lower cell abundances, growth rates and cellular particulate organic contents were observed compared to 13°C for both 400 μatm and 800 μatm pCO_2_ conditions (Fig 4A–4C). *C*. *socialis* species are widely distributed in the world oceans and are commonly found from the cold waters of the Arctic, the North Atlantic to the much warmer waters of the Mediterranean Sea or the Gulf of California covering a wide temperature range from -2 to 29°C [74, 75]. However, a clear physiological difference was found between the northern (isolated from NE Atlantic/Arctic) and the southern (isolated from Mediterranean Sea) groups of *C*. *socialis* strains at 2.5, 8 and 13°C [76, 77]. At lower temperatures (< 13°C), the northern strains showed significantly higher growth rates, cell densities and photosynthetic yields compared to the southern ones. Accordingly, the biological response of *C*. *socialis* strain to temperature probably depends on its geographical distribution, demonstrating the considerable ecological plasticity of diatom *C*. *socialis* [76]. Our data strongly indicate that the northern *C*. *socialis* strains isolated from the Belgian Coast of the North Sea, where the annual mean surface temperature was around 12.5°C in the past 10 years, would favour lower temperature conditions at 13°C compared to 18°C. Thus, our findings on the effect of temperature can only be generalised for the northern strains, but not for all the strains of *C*. *socialis*.

In addition, a lower cellular POC content was observed at 18°C for both 400 μatm and 800 μatm pCO_2_ levels (Fig 4C). The POC content is basically proportional to the cell volume. Menden-Deuer and Lessard [78] proposed the following conversion equation: C = 0.288*V^0.811^ for diatoms (C denotes organic carbon content in pg C cell^-1^, and V represents the cell volume in μm^3^). It has been suggested that temperature has the potential to affect phytoplankton size, diminishing the cell size under high temperature conditions. De Bodt *et al*. [79] observed a reduction in coccosphere size of the calcifier *Emiliania huxleyi* with an increase of temperature from 13°C to 18°C. Smaller diatoms are believed to have the advantage of living under warmer circumstances because of a larger surface area to volume quotient [80]. Some field studies have also found that ocean warming is giving rise to a decline in phytoplankton cell size, a shift toward a phytoplankton assemblage dominated by smaller phytoplankton [81, 82]. Furthermore, changes in temperature will lead to the shifts in the geographical distributions of marine species (e.g., towards higher latitudes or deeper waters). Pelagic phytoplankton can respond rapidly to changing environment, whose distribution may shift poleward by hundreds of kilometers per decade [21].

In conclusion, ocean warming may lead to a decline in global phytoplankton biomass and productivity and result in the shifts in the distribution and composition of different marine phytoplankton communities, which will finally alter the basis of the marine food web, biodiversity and regional ecosystems.

In our study, diatom *C*. *socialis* exhibited negative biological responses to elevated pCO_2_ conditions. At 800 μatm, cellular biomass, growth rate and cellular particulate organic content were found to be lower compared to 400 μatm for both 13°C and 18°C levels (Fig 4A–4C). However, due to the great variety of *Chaetoceros* species, *Chaetoceros sp*. shows complex and species-specific responses to ocean acidification. Enhanced, inhibited or absence of a significant impact on the growth of different *Chaetoceros* species has been reported in the literature [83–85]. Thus, the unfavourable responses to ocean acidification observed in our study cannot be generalized even for *Chaetoceros* species.

The diversity in responses to ocean acidification could be largely explained by the varied energetic efficiency and regulation of Carbon Concentrating Mechanisms (CCMs) among phytoplankton clades. Phytoplankton can acquire inorganic C through the C fixing enzyme RuBisCO which is catalytically inefficient and has a poor affinity for CO_2_. Therefore, marine diatoms as well as other phytoplankton taxa generate the energy-costly CCMs to enrich CO_2_ at the catalytic site of RubisCO and alleviate the CO_2_ limitation [86, 87]. Theoretically, photosynthesis and growth of diatoms can be limited by the availability of CO_2_. In view of the ongoing ocean acidification, the elevated CO_2_ concentrations may cause a down-regulation of the CCM capacity. Doubling of the atmospheric CO_2_ level would save about 20% of the energy demand for CCMs of diatoms [88]. From this point of view, the energy saved from the down-regulated CCMs of phytoplankton may stimulate their growth and enhance the oceanic primary production. However, the discrepancies in the responses to ocean acidification between diatom species indicate that the energy saved due to the down-regulated CCM may not always be reallocated to support diatoms growth. It may depend on the balance between the simulative and negative effects associated with increased CO_2_ and seawater acidification. For instance, Wu *et al*. [84] found that the saved energy could increase the growth rate of diatom *Phaeodactylum tricornutum* by 5% when exposed to 1000 μatm pCO_2_. While for the same diatom, the mitochondrial respiration also increased and photosynthetic electron transport was inhibited because of the CO_2_-induced seawater acidification, which could eventually offset the reserved energy from down regulation of CCMs activity.

Additionally, as shown in Fig 4c, a lower cellular POC content was observed at 800 μatm pCO_2_ for both 13°C and 18°C levels (Fig 4C). It implies that CO_2_ concentrations, like temperature, can also influence the phytoplankton size, by decreasing cell size with increasing pCO_2_. Our findings may suggest an increasing fraction of smaller phytoplankton in warmer and less alkaline waters. A decline in cell size in response to high pCO_2_ conditions has been reported for micro- and nano-sized phytoplankton such as the diatom *Amphora coffeaeformis* and *Nitzschia ovalis* [80] and the coccolithophore *Emiliania huxleyi* [79]. Moreover, previous studies have reported significant changes in primary productivity and phytoplankton community structure in response to increased pCO_2_ [89, 90]. Tortell *et al*. [89] reported a decline in the abundance of *Phaeocystis sp*. and an increase in diatom biomass at high pCO_2_ (750 μatm) in the eastern Subtropical and Equatorial Pacific Ocean. Hare *et al*. [90] reported that diatoms were replaced by autotrophic nanoflagellates at high pCO_2_ (750 μatm) in the Bering Sea.

Despite the controversial and contradictory conclusions obtained in these studies, substantial evidence has already accumulated and indicated that CO_2_-related changes in seawater and thus ocean acidification can directly affect marine phytoplankton. They range from physiological responses on the organism level to the changes in the community composition and structure.

### 4.3 The effects of climate change on nutrients cycles

Marine environmental conditions are changing rapidly, particularly the modifications in seawater temperature and pH induced by ocean warming and acidification. They have far-reaching significances on marine biogeochemical cycles of C and nutrients, such as phytoplankton stoichiometric composition C:N:P, Si and biologically essential trace metal Fe.

The Redfield C:N:P ratio of 106:16:1 is widely used as an average composition of these elements in phytoplankton [91]. In our research, no considerable shifts in the C:N:P ratio were observed during the exponential growth phase of diatom *C*. *socialis* for each of the four treatments (Figs 5 and 6). In Fig 5, almost all the data points of POC/PN ratio lie along the line corresponding to the Redfield C:N ratio of 6.6. In Fig 6A, a large variation of ΔN/ΔP drawdown ratios was observed but the overall mean ratios were 17 close to the Redfield N:P ratio of 16. However, considerable changes in C:N:P composition in response to increasing pCO_2_ and temperature were observed. During the mesocosm experiments, Bellerby *et al*. [92] found C:N:P stoichiometry of organic production in a pelagic phytoplankton assemblage increased approximately from 121:6:1 to 144:7:1 to 168:8:1 when pCO_2_ was raised from 350 to 700 to 1050 μatm. King *et al*. [93] tested the effects of CO_2_ on elemental composition C:N:P using 7 wide-spread coastal and oceanic phytoplankton species. Among these species, rising CO_2_ availability led to higher C:P and N:P ratios in one species (~60 to 90% higher), lower C:P and N:P ratios in 3 species (~20 to 50% lower), and no significant change in 3 species. The influence of temperature on C:N:P ratios is supposed to be negligible, even though temperature will indeed affect both metabolism and physico-chemical interactions between phytoplankton cells and the environment [94].

Diatom cells are covered within a unique silica cell well known as frustule. Because Si is essential for diatom growth, diatoms are believed to play a key role in the regulation of the biogeochemical cycle of Si in the modern ocean. Diatoms have an average N/Si elemental ratio of 1, although this ratio has been found to be highly variable ranging from 0.14 to 5.88 depending on the conditions of light, temperature, and nutrient availability [95]. In Fig 6b, nearly all the data points of ΔN/ΔSi drawdown ratio were above the line of the regular N/Si ratio of 1. The mean ΔN/ΔSi drawdown ratios observed during the exponential growth phase for the four treatments were 3.8, three times higher than the average. It should be noted that large deviations of elemental composition ratio are generally found in phytoplankton growing in nutrient limited waters under conditions of unbalanced nutrient supply [96]. Through the preliminary tests, we found that our desert dust could provide additional PO4 and Si, but not NO3 for diatom growth. So we had to ensure that NO3 was not limited throughout the experiment, otherwise the diatom growth would not be boosted after dust addition. Our results exhibited a higher N/Si elemental ratio than the average value because the strains were exposed to the Si-limited conditions relative to N.

Seawater pH and temperature, as two most basic and important variables in all chemical and biological processes, could alter Fe bioavailability through changing solubility of Fe-containing minerals, organic complexation, Fe redox chemistry, and dust dissolution processes [97]. In response to the dust addition, our results demonstrated a two-fold increase in dissolved Fe^3+^ concentrations for the high CO_2_ treatment (800 μatm) in comparison to the low CO_2_ condition (400 μatm), following a pH reduction from 8.04 to 7.75 (Fig 3B). However, no significant difference in dissolved Fe^2+^ concentrations was observed (Fig 3A, a one-way ANOVA, p>0.05). When seawater pH drops below 8, shifts in the inorganic speciation of Fe will lead to an increase in the thermodynamic solubility of Fe^3+^ (oxy)hydroxide. Meanwhile, ocean acidification will slow down the oxidation rate of Fe^2+^ and thus increase the residence time of Fe^2+^ [8]. Millero *et al*. [98] suggested an increase in Fe solubility of 40% with a pH decrease from 8.1 to 7.4. Breitbarth *et al*. [3] conducted a mesoscale CO_2_ enrichment experiment and found that ocean acidification could significantly increase the fraction of dissolved Fe^3+^ and Fe^2+^ and thus enhance Fe bioavailability. On the contrary, based on both laboratory and field experiments, Shi *et al*. [99] observed a reduction in Fe bioavailability and phytoplankton uptake in response to ocean acidification. For the temperature effect on the dissolved Fe release through dust addition, there was no observed statistical variance at 13°C and 18°C (Fig 3). In general, the inorganic solubility of Fe decreases with increasing temperature [97]. However, the organic solubility of ligand-bound Fe may probably not change with temperature [100]. Ultimately, the impact of temperature on dissolved Fe availability depends on the interaction of inorganic and organic forms in seawater. Hence, the influence of ocean acidification and warming on Fe biogeochemistry depends not only on pH and/or temperature, but also on a complex interrelationship of inorganic solubility, organic complexation, redox chemistry, and the phytoplankton-trace metal feedback mechanisms [101].

## 5. Conclusions

In the light of our laboratory culture studies, diatom C. *socialis* was found to be sensitive to climate-induced changes, such as dust deposition, and ocean warming and acidification. Our results will require further confirmation, but they suggest that 1) Desert dust particles can provide macronutrients (PO4 and DSi) and dissolved Fe, and could thus significantly enhance the diatom growth and production in PO4- and DSi-deprived waters, mainly through the release of these two nutrients; 2) Ocean acidification can help the release of Fe upon dust deposition; 3) Elevated pCO_2_ and thus ocean acidification may limit the growth of diatom C. *socialis*, reduce their cell size and affect the phytoplankton composition; 4) Warming may also have adverse impact on diatom C. *socialis* growth, reducing their cell size and causing their poleward shifts in biogeographic distribution; and 5) Ocean acidification and warming may have an interactive effects and further constrain the growth of diatom C. *socialis* in the future oceans, which could render C. *socialis* less competitive over other phytoplankton. Predicted negative biological responses of some phytoplankton species to changing climate may affect phytoplankton growth, population size, biogeographic distribution, net primary productivity, community composition, marine food webs, and nutrient limitation pattern and biogeochemical cycles.

In order to comprehend how global climate change will further impact phytoplankton in the future, we need to conduct more extensive research to improve our understanding in the following aspects: 1) Combined or interactive effects of multiple environmental variables, in combination of CO_2_, temperature, dust deposition, light and nutrient availability *etc*.; 2) Multifarious taxon-, species- and strain-specific responses to climate change; 3) Potential shifts in phytoplankton community composition in response to changing climate through on-board incubation or mesocosms experiments; 4) Prospective long-term physiological acclimatization or evolutionary adaptation in order to keep pace with the rate of climate change.

## Supporting information

S1 Fig Evolution of diatom growth and nutrient concentrations during the culture experiments at 400 μatm pCO_2_ and 13°C. (A) chlorophyll-a, (B) cell number, (C) NO3, (D) PO4, (E) DSi and BSi before dust addition on day 14 (reaching maximum growth), (F) DSi after dust addition on day 14, (G) dissolved Fe^+2^, (H) dissolved Fe^+3^, (I) POC and (J) PN for both treatments, without and with dust addition. No dust addition treatment, before and after dust addition treatments and dust dissolution test are shown respectively as open circles, closed black and grey circles, and open diamonds. Vertical dashed lines indicate the day on which the dust (100 mg/L) was added to the dust treatment bottle. For (E), open and close triangles denote BSi concentrations in no dust treatment and dust treatment, respectively.
(TIF)

S2 Fig Evolution of diatom growth and nutrient concentrations during the culture experiments at 800 μatm pCO_2_ and 13°C. (A) chlorophyll-a, (B) cell number, (C) NO3, (D) PO4, (E) DSi and BSi before dust addition on day 14 (reaching maximum growth), (F) DSi after dust addition on day 14, (G) dissolved Fe^+2^, (H) dissolved Fe^+3^, (I) POC and (J) PN for both treatments, without and with dust addition. No dust addition treatment, before and after dust addition treatments and dust dissolution test are shown respectively as open circles, closed black and grey circles, and open diamonds. Vertical dashed lines indicate the day on which the dust (100 mg/L) was added to the dust treatment bottle. For (E), open and close triangles denote BSi concentrations in no dust treatment and dust treatment, respectively.
(TIF)

S3 Fig Evolution of diatom growth and nutrient concentrations during the culture experiments at 800 μatm pCO_2_ and 18°C. (A) chlorophyll-a, (B) cell number, (C) NO3, (D) PO4, (E) DSi and BSi before dust addition on day 14 (reaching maximum growth), (F) DSi after dust addition on day 14, (G) dissolved Fe^+2^, (H) dissolved Fe^+3^, (I) POC and (J) PN for both treatments, without and with dust addition. No dust addition treatment, before and after dust addition treatments and dust dissolution test are shown respectively as open circles, closed black and grey circles, and open diamonds. Vertical dashed lines indicate the day on which the dust (100 mg/L) was added to the dust treatment bottle. For (E), open and close triangles denote BSi concentrations in no dust treatment and dust treatment, respectively.
(TIF)
