# Supplementary figures and images for: Biological responses of the marine diatom Chaetoceros socialis to changing environmental conditions: A laboratory experiment
Source: PLoS One. 2017 Nov 30;12(11):e0188615. doi: 10.1371/journal.pone.0188615 (PMC5708725; doi:10.1371/journal.pone.0188615)

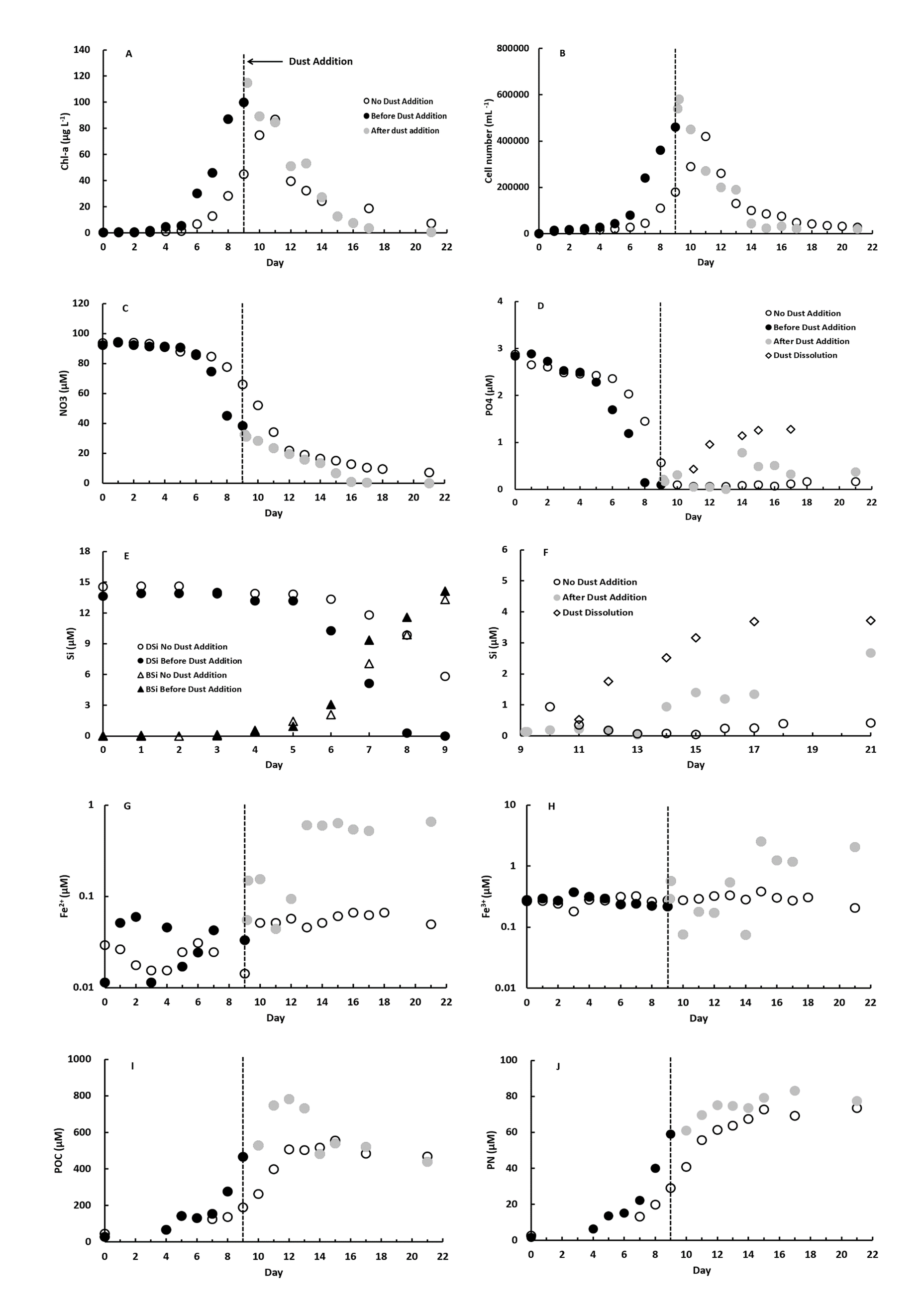

Supplement: S1 Fig — (A) chlorophyll-a, (B) cell number, (C) NO3, (D) PO4, (E) DSi and BSi before dust addition on day 14 (reaching maximum growth), (F) DSi after dust addition on day 14, (G) dissolved Fe+2, (H) dissolved Fe+3, (I) POC and (J) PN for both treatments, without and with dust addition. No dust addition treatment, before and after dust addition treatments and dust dissolution test are shown respectively as open circles, closed black and grey circles, and open diamonds. Vertical dashed lines indicate the day on which the dust (100 mg/L) was added to the dust treatment bottle. For (E), open and close triangles denote BSi concentrations in no dust treatment and dust treatment, respectively. (TIF) [file pone.0188615.s001.tif]

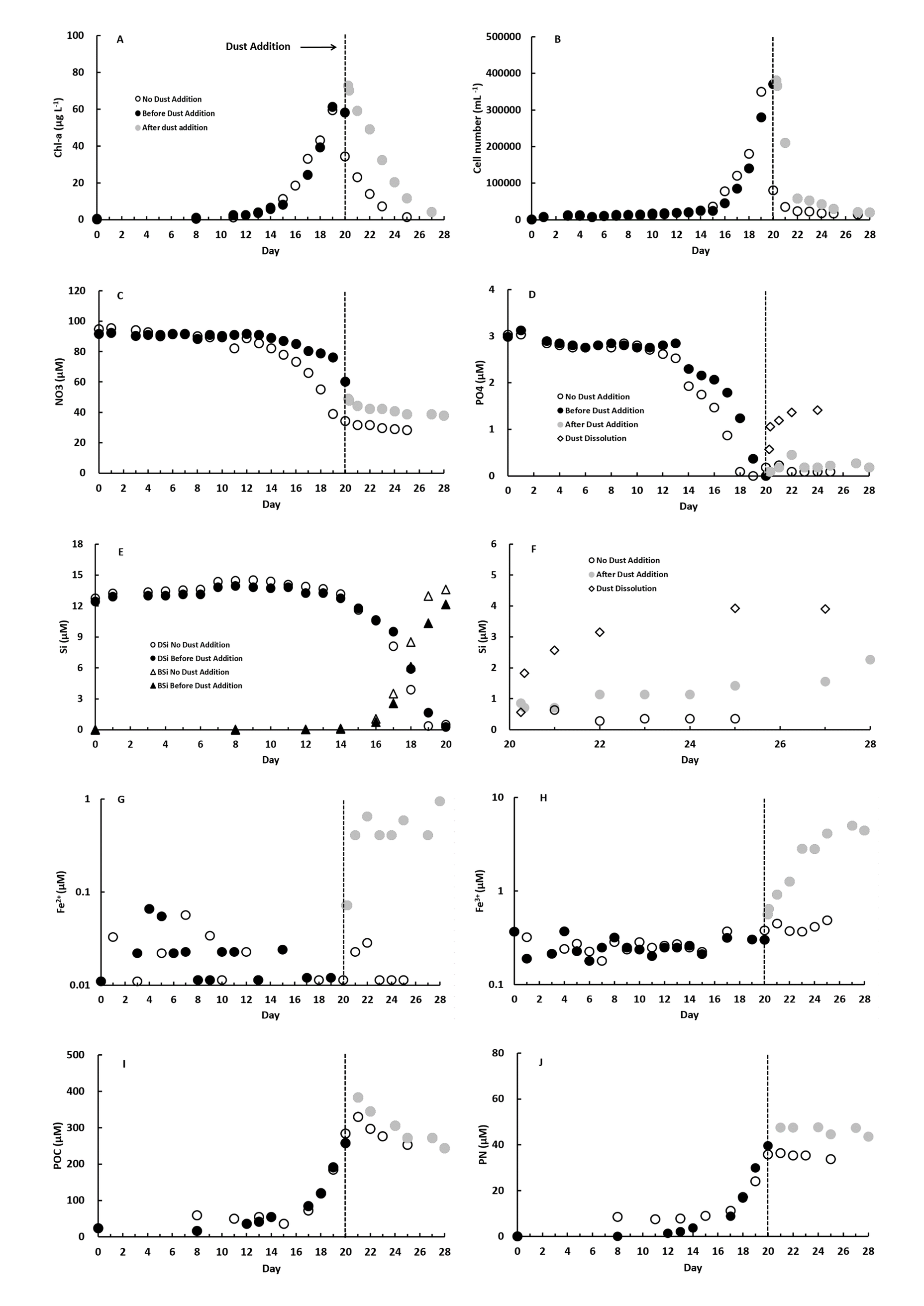

Supplement: S2 Fig — (A) chlorophyll-a, (B) cell number, (C) NO3, (D) PO4, (E) DSi and BSi before dust addition on day 14 (reaching maximum growth), (F) DSi after dust addition on day 14, (G) dissolved Fe+2, (H) dissolved Fe+3, (I) POC and (J) PN for both treatments, without and with dust addition. No dust addition treatment, before and after dust addition treatments and dust dissolution test are shown respectively as open circles, closed black and grey circles, and open diamonds. Vertical dashed lines indicate the day on which the dust (100 mg/L) was added to the dust treatment bottle. For (E), open and close triangles denote BSi concentrations in no dust treatment and dust treatment, respectively. (TIF) [file pone.0188615.s002.tif]

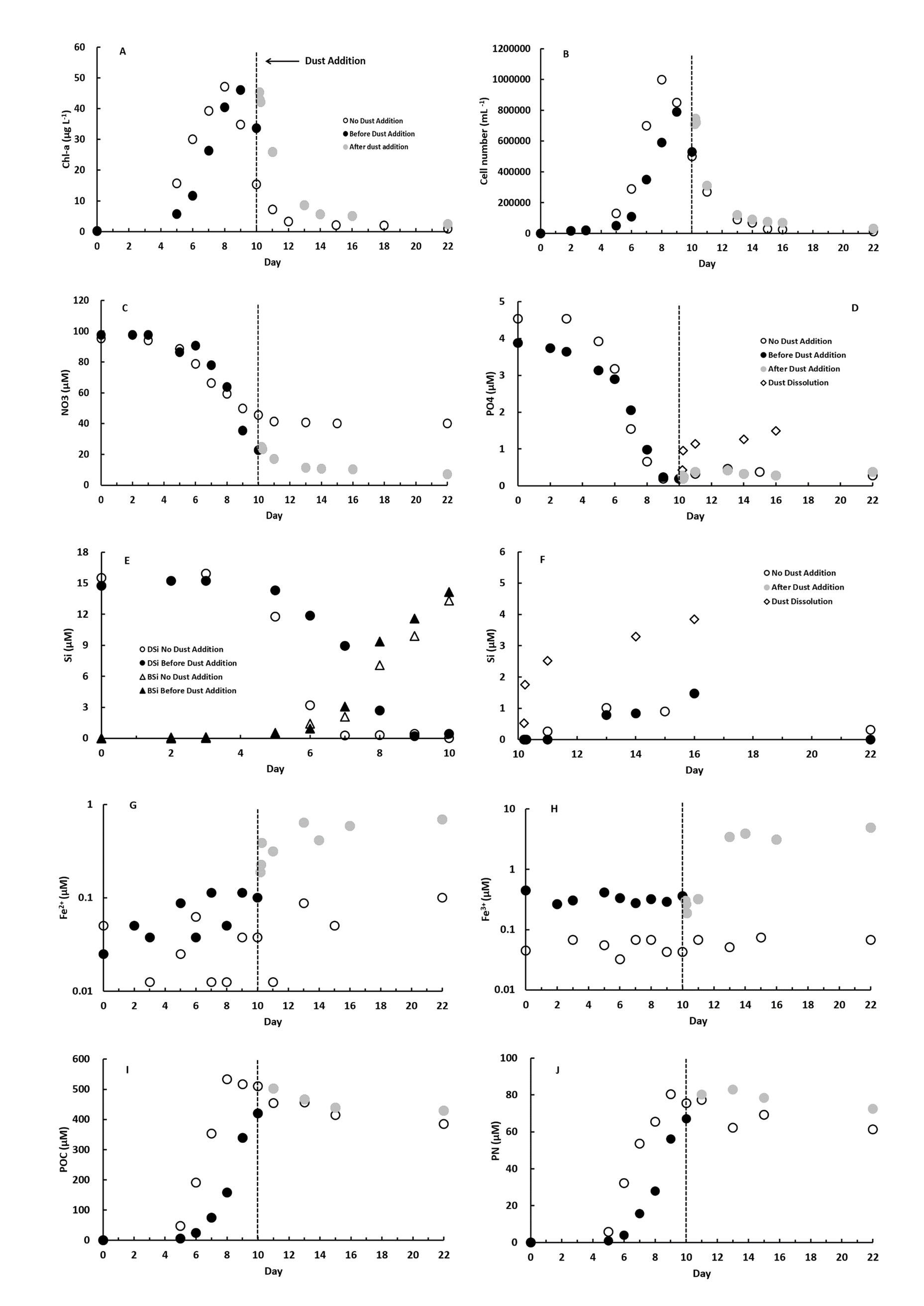

Supplement: S3 Fig — (A) chlorophyll-a, (B) cell number, (C) NO3, (D) PO4, (E) DSi and BSi before dust addition on day 14 (reaching maximum growth), (F) DSi after dust addition on day 14, (G) dissolved Fe+2, (H) dissolved Fe+3, (I) POC and (J) PN for both treatments, without and with dust addition. No dust addition treatment, before and after dust addition treatments and dust dissolution test are shown respectively as open circles, closed black and grey circles, and open diamonds. Vertical dashed lines indicate the day on which the dust (100 mg/L) was added to the dust treatment bottle. For (E), open and close triangles denote BSi concentrations in no dust treatment and dust treatment, respectively. (TIF) [file pone.0188615.s003.tif]
